# Glycerol enhances fungal germination at the water‐activity limit for life

**DOI:** 10.1111/1462-2920.13530

**Published:** 2016-11-13

**Authors:** Andrew Stevenson, Philip G. Hamill, Ángel Medina, Gerhard Kminek, John D. Rummel, Jan Dijksterhuis, David J. Timson, Naresh Magan, Su‐Lin L. Leong, John E. Hallsworth

**Affiliations:** ^1^ Institute for Global Food Security, School of Biological Sciences, MBC, Queen's University BelfastBelfastBT9 7BLNorthern Ireland; ^2^ Applied Mycology Group, Cranfield Soil and AgriFood Institute, Cranfield UniversityCranfieldBedfordMK43 OALUK; ^3^ Independent Safety Office, European Space Agency2200 AG NoordwijkThe Netherlands; ^4^ SETI InstituteMountain ViewCalifornia94043USA; ^5^ CBS‐KNAW Fungal Biodiversity CentreUppsalalaan 8UtrechtCT3584The Netherlands; ^6^ School of Pharmacy and Biomolecular SciencesUniversity of BrightonHuxley Building, Lewes RoadBrightonBN2 4GJUK; ^7^ Department of MicrobiologySwedish University of Agricultural SciencesBox 7025Uppsala75007Sweden

## Abstract

For the most‐extreme fungal xerophiles, metabolic activity and cell division typically halts between 0.700 and 0.640 water activity (approximately 70.0–64.0% relative humidity). Here, we investigate whether glycerol can enhance xerophile germination under acute water‐activity regimes, using an experimental system which represents the biophysical limit of Earth's biosphere. Spores from a variety of species, including *Aspergillus penicillioides*, *Eurotium halophilicum*, *Xerochrysium xerophilum* (formerly *Chrysosporium xerophilum*) and *Xeromyces bisporus*, were produced by cultures growing on media supplemented with glycerol (and contained up to 189 mg glycerol g dry spores^−1^). The ability of these spores to germinate, and the kinetics of germination, were then determined on a range of media designed to recreate stresses experienced in microbial habitats or anthropogenic systems (with water‐activities from 0.765 to 0.575). For *A. penicillioides*, *Eurotium amstelodami*, *E. halophilicum*, *X. xerophilum* and *X. bisporus*, germination occurred at lower water‐activities than previously recorded (0.640, 0.685, 0.651, 0.664 and 0.637 respectively). In addition, the kinetics of germination at low water‐activities were substantially faster than those reported previously. Extrapolations indicated theoretical water‐activity minima below these values; as low as 0.570 for *A. penicillioides* and *X. bisporus*. Glycerol is present at high concentrations (up to molar levels) in many types of microbial habitat. We discuss the likely role of glycerol in expanding the water‐activity limit for microbial cell function in relation to temporal constraints and location of the microbial cell or habitat. The findings reported here have also critical implications for understanding the extremes of Earth's biosphere; for understanding the potency of disease‐causing microorganisms; and in biotechnologies that operate at the limits of microbial function.

## Introduction

Glycerol, which can be present in the extracellular environment or within the cytosol at high concentrations, is a recurring motif in the physiology of extremophilic microbes. It is fungal xerophiles such as *Aspergillus penicillioides* and *Xeromyces bisporus* which dominate league tables for ability to grow in high‐solute environments and/or at low water‐availability (Stevenson *et al*., 2015a); these achievements can, in part, be attributed to their ability to accumulate and retain extraordinary levels of glycerol for osmotic adjustment. Along with some other microbes, these fungi are both capable of accumulating glycerol and are commonly associated with environments where glycerol reaches molar concentrations, including saline and sugar‐rich habitats; various types of fermentation milieu; foods, feeds and other manufactured products and within experimental systems (Hallsworth and Magan, 1994b; 1995; Wang *et al*., 2001; Patiño‐Vera *et al*., 2005; Bardavid *et al*., 2008; Basso *et al*., 2008; Donkin, 2008; Williams and Hallsworth, 2009; Chin *et al*., 2010; de Lima Alves *et al*., 2015; Lievens *et al*., 2015; Leong *et al*., 2015; Santos *et al*., 2015; Stevenson *et al*., 2015a). For instance, cells of fungi and algae can contain 7–8 M glycerol (see below); high intracellular glycerol is a determinant for vigour (Hallsworth and Magan, 1994a, 1995; de Jong *et al*., 1997); and the insect haemolymph, in which entomopathogenic fungi proliferate, can also contain glycerol at molar concentrations (Sformo *et al*., 2010). Studies of bacteria and fungi are carried out on culture‐media in the range 4–8 M glycerol (e.g. Santos *et al*., 2015; Stevenson *et al*., 2015a); and glycerol can also accumulate as a product in industrial systems (Wang *et al*., 2001; Cray *et al*., 2015a). Whereas *in vitro* studies of microbial solute stress typically focus on individual stressors, single‐solute systems are unrepresentative of extreme habitats found in nature (e.g. Lievens *et al*., 2015; Stevenson *et al*., 2015a; Yakimov *et al*., 2015). A recent study of extreme halophilic bacteria and Archaea, previously thought to have a 0.755 water‐activity limit for growth and metabolism (Grant, 2004; Kminek *et al*., 2010; Rummel *et al*., 2014), revealed cell division at 0.635 water activity with a theoretical minimum of 0.611 water activity, for cultures in mixed‐solute substrates (Stevenson *et al*., 2015a). Almost 70 years ago, a study of fungal xerophiles established a water‐activity limit of 0.640 for germination of *Eurotium echinulatum* conidia (a value equivalent to 64.0% equilibrium relative humidity); though the germination process was severely inhibited: germ‐tube formation only occurred after a 2‐year incubation period (Snow, 1949). Snow (1949) also reported evidence of a low level of (aborted) germination below this value: ‘One or two conidia … produced germ tubes at [0.620 water activity, though] many of the germ tubes produced were misshapen and probably not viable’. Other studies have reported germination for spores of *X. bisporus*, *A. penicilloides*, *Xerochrysium xerophilum* and other species in the range 0.740–0.700 which failed to yield any subsequent development of mycelium (Gock *et al*., 2003). Pitt and Christian (1968) reported limits of 0.644 and 0.605 water activity for germination of *X. bisporus* ascospores and aleuriospores respectively (though neither the authors of the original study nor ourselves have been able to repeat the aleuriospore study; data not shown).

It is well‐established that temperature can impact the water‐activity minima for microbial growth; a series of recent studies has demonstrated that chaotropicity can also modify microbial water relations. Indeed, concentrations and proportion of chaotropic and kosmotropic solutes can determine biotic activity within both saline and non‐saline habitats (Hallsworth *et al*., 2007; Williams and Hallsworth, 2009; Chin *et al*., 2010; Cray *et al*., 2015a; Lievens *et al*., 2015; de Lima Alves *et al*., 2015; Stevenson *et al*., 2015b; Yakimov *et al*., 2015). However, there is a considerable knowledge gap in relation to the microbiology of glycerol. Few studies have focused on glycerol as a determinant for the limits for life (Williams and Hallsworth, 2009; Stevenson *et al*., 2015a; in press); there is paucity of information on the ecophysiology of fungal germination in relation to the solute composition of high‐glycerol milieu; and there has been no systematic study of microbial germination in relation to the biophysical activities (e.g. chao‐/kosmotropicity) of any of the other solutes known to regulate cellular and ecosystem function (e.g. Cray *et al*., 2013a; Oren and Hallsworth, 2014; Stevenson *et al*., 2015a; Wyatt *et al*., 2015; Yakimov *et al*., 2015).

This study was carried out, taking inspiration from the natural ecology of extremophiles, to investigate whether glycerol can determine biotic activity at the water‐activity limit for life. A dual approach was used: extreme xerophilic fungi were encouraged to produce spores which accumulated glycerol, following which these propagules were assayed for ability to germinate on high‐glycerol substrates. These germination assays were carried out in the range 0.765–0.575 water activity, i.e. at biologically hostile water activities, for seven fungal species of the order Eurotiales. They were: *A. penicillioides* (three strains), *Eurotium amstelodami*, *E. echinulatum*, *Eurotium halophilicum* and *Eurotium repens* (all in the *Aspergillus sensu stricto*, Houbraken and Samson, 2011) and the closely related species *X. bisporus* (four strains) and *Xerochrysum xerophilum* (formerly *Chrysosporium xerophilum*). We hypothesized that (i) composition of biophysical stressors within a fungal substrate determines ability to germinate at low water‐activity; and glycerol can both (ii) speed up the kinetics of germination and (iii) enable germination at hitherto unprecedented water activities.

## Results and discussion

### Accumulation of glycerol within spores of extreme xerophiles

Intracellular accumulation of glycerol enhances stress tolerance for both spores and mycelium which are exposed to low water‐activity, osmotic stress, chaotropicity, hydrophobic substances, salt‐induced stresses and other challenges (e.g. Hallsworth and Magan, 1995; Hallsworth *et al*., 2003b; Bhaganna *et al*., 2010).^1^ Glycerol can also play roles in pathogenic processes and other trophic interactions (Hallsworth and Magan, 1994a, 1995, 1995; de Jong *et al*., 1997, Cray *et al*., 2013a; Paulussen *et al*., in press). In relation to germination processes, spores containing 2.8–8.5% w/v glycerol are known to germinate more vigorously, at lower water‐activity and (for pathogenic fungi) exhibit higher levels of virulence (Hallsworth and Magan, 1994a, 1995, 1995; Cray *et al*., 2013a; Hallsworth *et al*., 2003b). For diverse fungi, the amount of intracellular glycerol in spores or hyphae is inversely proportional to the water activity of the substrate (Hallsworth and Magan, 1995; de Lima Alves *et al*., 2015). We therefore supplemented media with a glycerol concentration 5.5 M (0.821 water‐activity), which is sufficiently high to promote the accumulation of glycerol as an osmolyte and yet moderate enough to facilitate substantial colony development. All strains produced spores aerially: conidia for *A. penicillioides*, ascospores and conidia for *Eurotium* strains (though only conidial germination of *Eurotium* species was assessed in this study; see *Experimental procedures*), and D‐shaped ascospores for *X. bisporus* strains (Pettersson *et al*., 2011). For further details of strain origin and biology see Supporting Information Table S1. Spores contained between 189 and 12.0 mg glycerol g dry spores^−1^, depending on strain (Supporting Information Fig. S1).^2^ High levels of glycerol have previously been reported in spores of entomopathogenic fungi; >90 mg glycerol g dry spores^−1^ (Hallsworth and Magan, 1994b, 1995). For physiologically active cells,, including germinating spores of xerophilic fungi, glycerol concentrations can be as high as 8 M (e.g. Hallsworth and Magan, 1994b; Bardavid *et al*., 2008; de Lima Alves *et al*., 2015).

### Biologically permissive versus biologically hostile culture‐media used for germination assays

High‐glycerol spores of each fungal strain were inoculated onto the 36 types of high‐glycerol culture medium (Supporting Information Table S2). These media were supplemented with glycerol + NaCl; glycerol + sucrose; glycerol + glucose +fructose; glycerol only; glycerol+ NaCl + sucrose or glycerol + NaCl + sucrose + KCl; representing various natural habitats and/or anthropogenic systems in which xerophiles are found (Andrews and Pitt, 1987; Dunman *et al*., 2001; Wang *et al*., 2001; Williams and Hallsworth, 2009; Bhaganna *et al*., 2010; Schubert *et al*. 2010; Kachalkin and Yurkov, 2012; Bennison and Karmanocky, 2014; Leong *et al*. 2015; Lievens *et al*., 2015; Rangel *et al*., 2015a; Stevenson *et al*., 2015a; in press; Oren, in press). For glycerol‐only media, the glycerol concentration varied between 7.0 and 7.7 M; all other media types were supplemented with 5.5 M glycerol plus additional solute(s), over a range of concentrations for the latter (Supporting Information Table S2). The 0.765–0.605 water‐activity range represents the tip of the biotic windows for growth or germination of the most extremely xerophilic strains (see Williams and Hallsworth, 2009; Stevenson *et al*., 2015a; in press). Previous studies have shown that it is glycerol, rather than mannitol, trehalose or other adaptations to the low water‐activity medium on which fungal spores were produced, which enhances germination of fungal propagules at low water‐activity or high chaotropicity (Hallsworth and Magan, 1995; Hallsworth *et al*., 2003b). The same finding was reported for high‐glycerol cells of the bacterium *Pseudomonas putida* which were exposed to benzene stress (Bhaganna *et al*., 2010); these findings are also consistent with the vigorous growth phenotypes observed on high‐glycerol substrates (Williams and Hallsworth, 2009; Stevenson *et al*., 2015a). Furthermore, glycerol is the only compatible solute which is sufficiently soluble to reduce intracellular water activity to levels significantly below 0.650; other polyols for instance cannot facilitate osmotic adjustment for the water‐activity range used in this study, i.e. ≤0.765 (Hallsworth and Magan, 1995; de Lima Alves *et al*., 2015). Glycerol is also superior to other organic compatible solutes in its ability to accumulate to high molar concentrations (like xerophiles, phylogenetically diverse halophiles can accumulate glycerol to ≥7 M) and reduce intracellular water‐activity to below the known limits for microbial life.

**Figure 1:**
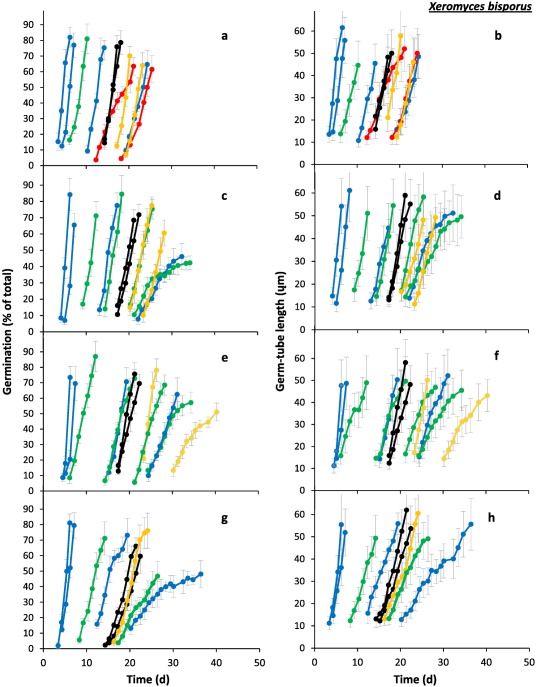
Progress of spore germination for four strains of *X. bisporus*: (**a** and **b**) FRR 0025; (**c** and **d**) FRR 1522; (**e** and **f**) FRR 2347 and (**g** and **h**) FRR 3443. Percentage germination (**a**, **c**, **e** and **g**) and mean germ‐tube length (**b**, **d**, **f** and **h**) were determined on Malt‐Extract Yeast‐Extract Phosphate Agar (MYPiA) supplemented with diverse stressor(s) and incubated at 30°C for up to 50 days. Media were supplemented with: glycerol (red lines), at 7.0 and 7.1 M, with water‐activity values of 0.707 and 0.664, respectively; glycerol (5.5 M) + NaCl at 0.5, 1.0, 1.5 and 1.6 M (green lines), with water‐activity values of 0.765, 0.741, 0.709 and 0.692, respectively; glycerol (5.5 M) + sucrose at 0.25, 0.50, 0.65 and 0.80 M (blue lines), with water‐activity values of 0.734, 0.699, 0.674 and 0.637, respectively; glycerol (5.5 M) + NaCl (0.5 M) + sucrose at 0.3 and 0.5 M (black lines), with water‐activity values of 0.701 and 0.685, respectively; glycerol (5.5 M) + glucose (0.8 M) + fructose at 0.8 M and glycerol (5.5 M) + glucose (1.0 M) + fructose at 1.0 M (both grey lines), with water‐activity values of 0.694 and 0.649, respectively (see Supporting Information Table S2). For all media types, and regardless of fungal strain, germination occurred first at the highest water‐activity; for each medium type there are less individual media represented on this display than in Supporting Information Table S2, indicating that strains failed to germinate on the lower water‐activity media in the range. Grey bars indicate standard errors. For any colours which are not featured in the plots there was no germination observed for the corresponding range of media.

**Figure 2:**
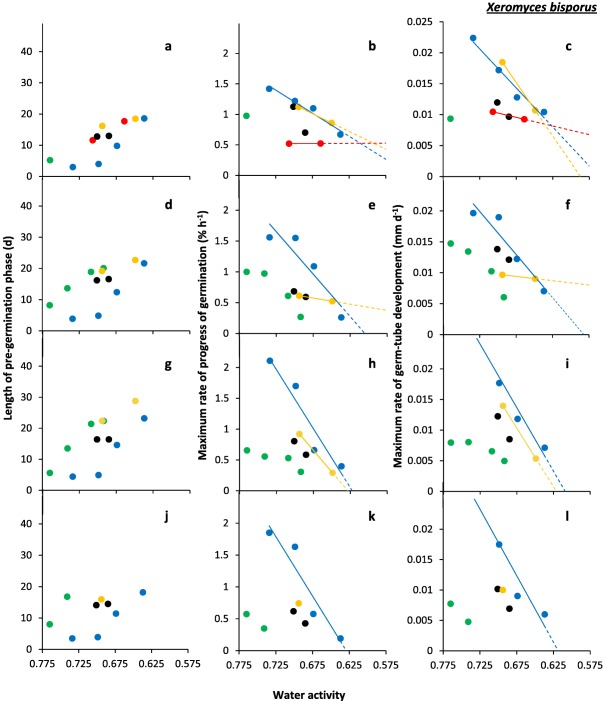
Kinetic profiles for germination of four strains of *X. bisporus*: (**a, b** and **c**) FRR 0025; (**d**, **e** and **f**) FRR 1522; (**g**, **h** and **i**) FRR 2347 and (**j**, **k** and **l**) FRR 3443. Length of the pre‐germination phase (**a**, **d**, **g** and **j**), maximum rate of spore germination (**b**, **e**, **h** and **k**), and maximum rate of germ‐tube development **c**, **f**, **i** and **l**) were determined on Malt‐Extract Yeast‐Extract Phosphate Agar (MYPiA) supplemented with diverse stressor(s) and incubated at 30°C for up to 50 days. Media were supplemented with: glycerol (red dots), at 7.0 and 7.1 M, with water‐activity values of 0.707 and 0.664, respectively; glycerol (5.5 M) + NaCl at 0.5, 1.0, 1.5 and 1.6 M (green dots), with water‐activity values of 0.765, 0.741, 0.709 and 0.692, respectively; glycerol (5.5 M) + sucrose at 0.25, 0.50, 0.65 and 0.80 M (blue dots), with water‐activity values of 0.734, 0.699, 0.674 and 0.637, respectively; glycerol (5.5 M) + NaCl (0.5 M) + sucrose at 0.3 and 0.5 M (black dots), with water‐activity values of 0.701 and 0.685, respectively; glycerol (5.5 M) + glucose (0.8 M) + fructose at 0.8 M and glycerol (5.5 M) + glucose (1.0 M) + fructose at 1.0 M (both grey dots), with water‐activity values of 0.694 and 0.649 respectively (see Supporting Information Table S2). Values for length of the pre‐germination phase were derived by extrapolation (see *Experimental procedures*), and maximum rates of germination and germ‐tube development were determined from the curves shown in Fig. 1. For any colours which are not featured in the plots, there was no germination observed for the corresponding range of media. Linear regression was used to determine lines of best fit, shown in the colour used for the appropriate medium range, which were used to derive theoretical water‐activity minima for germination for selected strains (see main text).

**Figure 3:**
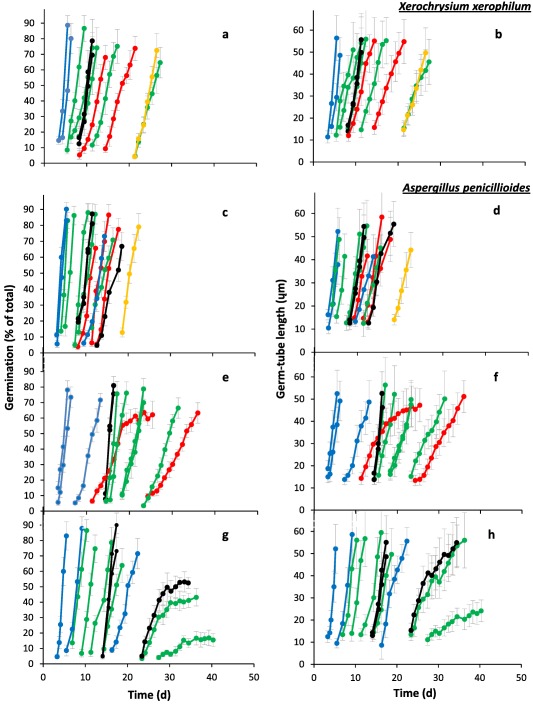
Progress of spore germination for *X. xerophilum*: (**a** and **b**) (FRR 0530) and three strains of *A. penicillioides*; (**c** and **d**) JH06GBM; (**e** and **f**) JH06THH and (**g** and **h**) JH06THJ). Percentage germination (**a**, **c**, **e** and **g**) and mean germ‐tube length (**b**, **d**, **f** and **h**) were determined on Malt‐Extract Yeast‐Extract Phosphate Agar (MYPiA) supplemented with diverse stressor(s) and incubated at 30°C for up to 50 days. Media were supplemented with: glycerol (red lines), at 7.0 and 7.1 M, with water‐activity values of 0.707 and 0.664, respectively; glycerol (5.5 M) + NaCl at 0.5, 1.0, 1.5 and 1.6 M (green lines), with water‐activity values of 0.765, 0.741, 0.709 and 0.692, respectively; glycerol (5.5 M) + sucrose at 0.25, 0.50, 0.65 and 0.80 M (blue lines), with water‐activity values of 0.734, 0.699, 0.674 and 0.637, respectively; glycerol (5.5 M) + NaCl (0.5 M) + sucrose at 0.3 and 0.5 M (black lines), with water‐activity values of 0.701 and 0.685, respectively; glycerol (5.5 M) + glucose (0.8 M) + fructose at 0.8 M and glycerol (5.5 M) + glucose (1.0 M) + fructose at 1.0 M (both grey lines), with water‐activity values of 0.694 and 0.649 respectively (see Supporting Information Table S2). For all media types, and regardless of fungal strain, germination occurred first at the highest water‐activity; for each medium type there are less individual media represented on this display than in Supporting Information Table S2, indicating that strains failed to germinate on the lower water‐activity media in the range. Grey bars indicate standard errors. For any colours which are not featured in the plots, there was no germination observed for the corresponding range of media.

**Figure 4:**
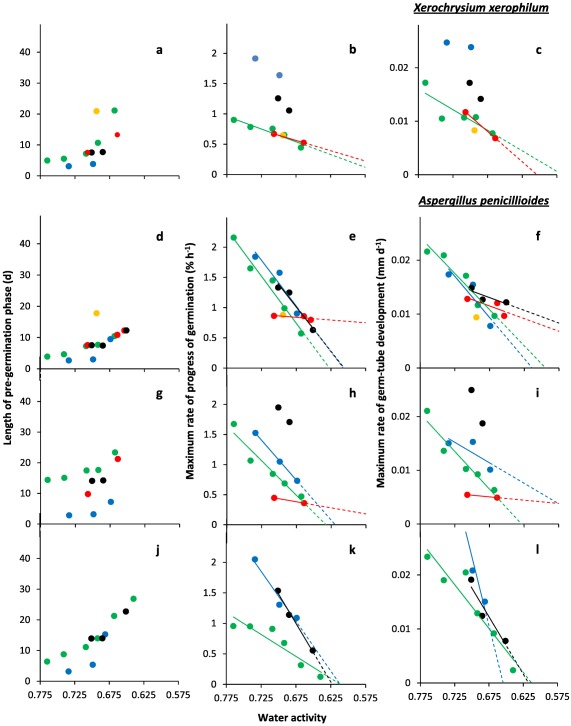
Kinetic profiles for germination of *X. xerophilum*; (**a**, **b** and **c**) (FRR 0530) and three strains of *A. penicillioides*; (**d**, **e** and **f**) JH06GBM; (**g**, **h** and **i**) JH06THH and (**j**, **k** and **l**) JH06THJ. Length of the pre‐germination phase (**a**, **d**, **g** and **j**), maximum rate of spore germination (**b**, **e**, **h** and **k**), and maximum rate of germ‐tube development (**c**, **f**, **i** and **l**) were determined on Malt‐Extract Yeast‐Extract Phosphate Agar (MYPiA) supplemented with diverse stressor(s) and incubated at 30°C for up to 50 days. Media were supplemented with: glycerol (red dots), at 7.0 and 7.1 M, with water‐activity values of 0.707 and 0.664, respectively; glycerol (5.5 M) + NaCl at 0.5, 1.0, 1.5 and 1.6 M (green dots), with water‐activity values of 0.765, 0.741, 0.709 and 0.692, respectively; glycerol (5.5 M) + sucrose at 0.25, 0.50, 0.65 and 0.80 M (blue dots), with water‐activity values of 0.734, 0.699, 0.674 and 0.637, respectively; glycerol (5.5 M) + NaCl (0.5 M) + sucrose at 0.3 and 0.5 M (black dots), with water‐activity values of 0.701 and 0.685, respectively; glycerol (5.5 M) + glucose (0.8 M) + fructose at 0.8 M and glycerol (5.5 M) + glucose (1.0 M) + fructose at 1.0 M (both grey dots), with water‐activity values of 0.694 and 0.649 respectively (see Supporting Information Table S2). Values for length of the pre‐germination phase were derived by extrapolation (see *Experimental procedures*), and maximum rates of germination and germ‐tube development were determined from the curves shown in Fig. 3. For any colours which are not featured in the plots, there was no germination observed for the corresponding range of media. Linear regression was used to determine lines of best fit, shown in the colour used for the appropriate medium range, which were used to derive theoretical water‐activity minima for germination for selected strains (see main text).

**Figure 5:**
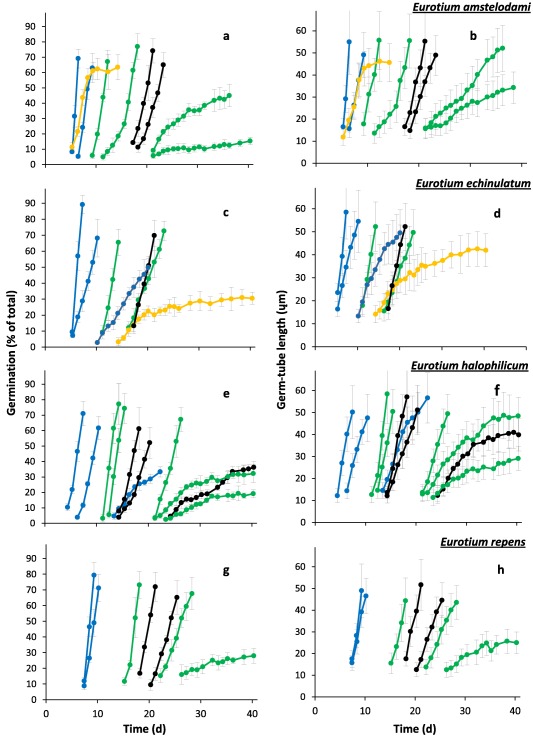
Progress of spore germination for *Eurotium amstelodami*; (**a** and **b**) (FRR 2792), *E. echinulatum*; (**c** and **d)** (FRR 5040), *E. halophilicum*; (**e** and **f**) (FRR 2471), and *E. repens*; (**g** and **h**) (JH06JPD). Percentage germination (**a**, **c**, **e** and **g**) and mean germ‐tube length (**b**, **d**, **f** and **h**) were determined on Malt‐Extract Yeast‐Extract Phosphate Agar (MYPiA) supplemented with diverse stressor(s) and incubated at 30°C for up to 50 days. Media were supplemented with: glycerol (red lines), at 7.0 and 7.1 M, with water‐activity values of 0.707 and 0.664, respectively; glycerol (5.5 M)+NaCl at 0.5, 1.0, 1.5 and 1.6 M (green lines), with water‐activity values of 0.765, 0.741, 0.709 and 0.692, respectively; glycerol (5.5 M) + sucrose at 0.25, 0.50, 0.65 and 0.80 M (blue lines), with water‐activity values of 0.734, 0.699, 0.674 and 0.637, respectively; glycerol (5.5 M) + NaCl(0.5 M) + sucrose at 0.3 and 0.5 M (black lines), with water‐activity values of 0.701 and 0.685, respectively; glycerol (5.5 M) + glucose (0.8 M) + fructose at 0.8 M and glycerol (5.5 M) + glucose (1.0 M) + fructose at 1.0 M (both grey lines), with water‐activity values of 0.694 and 0.649 respectively (see Supporting Information Table S2). For all media types, and regardless of fungal strain, germination occurred first at the highest water‐activity; for each medium type there are less individual media represented on this display than in Supporting Information Table S2, indicating that strains failed to germinate on the lower water‐activity media in the range. Grey bars indicate standard errors. For any colours which are not featured in the plots, there was no germination observed for the corresponding range of media.

**Figure 6:**
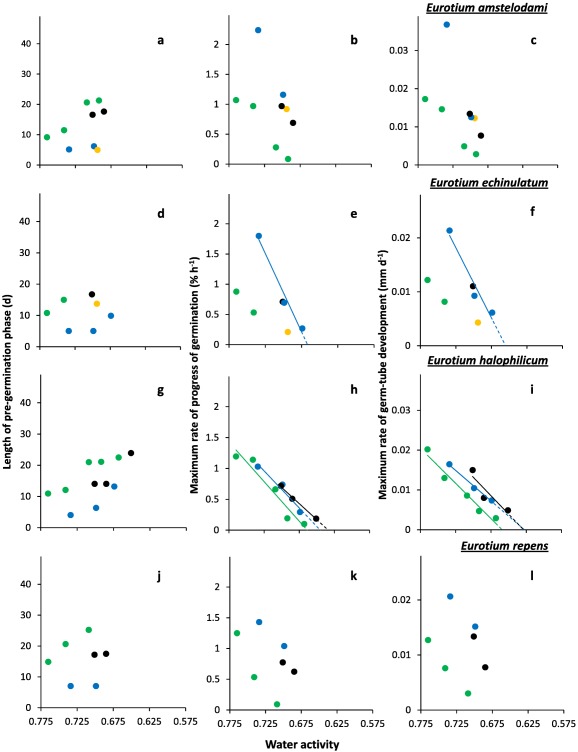
Kinetic profiles for germination of *E. amstelodami*; (**a**, **b** and **c**) (FRR 2792), *E. echinulatum*; (**d**, **e** and **f**) (FRR 5040), *E. halophilicum*; (**g**, **h** and **i**) (FRR 2471) and *E. repens*; (**j**,**k** and **h**) (JH06JPD). Length of the pre‐germination phase (**a**, **d**, **g** and **j**), maximum rate of spore germination (**b**, **e**, **h** and **k**), and maximum rate of germ‐tube development **c**, **f**, **i** and **l**) were determined on Malt‐Extract Yeast‐Extract Phosphate Agar (MYPiA) supplemented with diverse stressor(s) and incubated at 30°C for up to 50 days. Media were supplemented with: glycerol (red dots), at 7.0 and 7.1 M, with water‐activity values of 0.707 and 0.664, respectively; glycerol (5.5 M)+NaCl at 0.5, 1.0, 1.5 and 1.6 M (green dots), with water‐activity values of 0.765, 0.741, 0.709 and 0.692, respectively; glycerol (5.5 M) + sucrose at 0.25, 0.50, 0.65 and 0.80 M (blue dots), with water‐activity values of 0.734, 0.699, 0.674 and 0.637, respectively; glycerol (5.5 M) + NaCl (0.5 M) + sucrose at 0.3 and 0.5 M (black dots), with water‐activity values of 0.701 and 0.685, respectively; glycerol (5.5 M) + glucose (0.8 M) + fructose at 0.8 M and glycerol (5.5 M) + glucose (1.0 M)+fructose at 1.0 M (both grey dots), with water‐activity values of 0.694 and 0.649 respectively (see Supporting Information Table S2). Values for length of the pre‐germination phase were derived by extrapolation (see *Experimental procedures*), and maximum rates of germination and germ‐tube development were determined from the curves shown in Fig. 5. For any colours which are not featured in the plots, there was no germination observed for the corresponding range of media. Linear regression was used to determine lines of best fit, shown in the colour used for the appropriate medium range, which were used to derive theoretical water‐activity minima for germination for selected strains (see main text).

In this study, spores were sensitive to concentration and composition of stressors in the culture medium, regardless of xerophile strain (Supporting Information Table S2; Figs 1, 2, 3, 4, 5, 6). The strains able to germinate on the most types of culture media (i.e. all except glycerol + NaCl + sucrose + KCl) were *X. bisporus* FRR 0025, *X. xerophilum* FRR 0530 and *A. penicillioides* JH06GBM (Figs 1a and b; 4a–d respectively). By comparison, *A. penicillioides* JH06THJ; *E. halophilicum* FRR 2471 and *E. repens* JH06JPD were incapable of germination on any of the glycerol + glucose + fructose, glycerol‐only and glycerol + NaCl + sucrose + KCl media (Figs 3g and h; 5e–h). There was variation between both strains and species in relation to which media prohibited germination (see Stevenson *et al*., in press). Please note that both hyphal growth and germination of the xerophiles used in the current study are typically optimal in the range 0.930–0.830 (Williams and Hallsworth, 2009; Stevenson *et al*., 2015a). In this study, therefore, all compounds used to supplement media (which were at ≤0.765 water activity) can be properly regarded as stressors.

**Figure 7:**
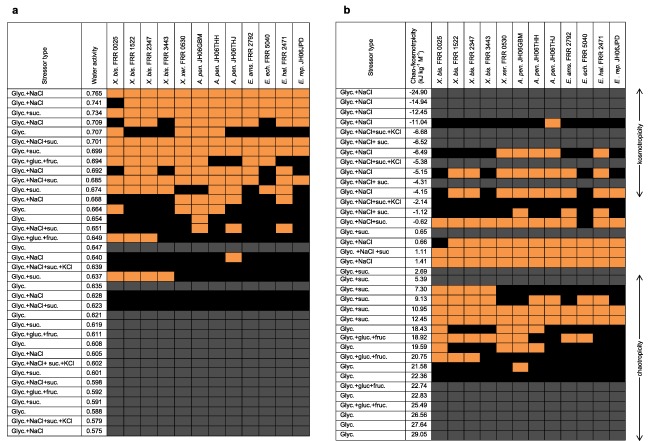
Culture media used for germination assays which permitted (orange) or prevented germination of xerophile strains (black and grey) in relation to their water activity **(a)** and chao‐/kosmotropicity **(b)**. For **(a)**, grey shading indicates media which were beyond the empirically determined chaotropicity limit for germination of these xerophiles (see main text); for **(b)**, grey shading indicates media which were beyond the empirically determined water‐activity limit for germination of these strains. Germination assays (see Figs 1, 2, 3, 4, 5, 6) were carried out on Malt‐Extract Yeast‐Extract Phosphate Agar (MYPiA) supplemented with diverse stressor(s) and incubated at 30°C for up to 50 days. For full names of media, and further details of their composition and properties, see Supporting Information Table S2.

*X. bisporus* FRR 0025 failed to germinate on glycerol + NaCl at ≤0.741 water activity (0.66 kJ g^−1^; i.e. chao‐/kosmotropicity neutral), glycerol + sucrose at ≤0.619 water activity (5.39 kJ g^−1^; i.e. mildly chaotropic), glycerol + glucose + fructose at ≤0.611 water activity (22.74 kJ g^−1^ chaotropic activity), glycerol only at ≤0.654 water activity (21.58 kJ g^−1^ chaotropic activity), glycerol + NaCl + sucrose at ≤0.651 water activity (−1.12 kJ g^−1^; i.e. chao‐/kosmotropicity neutral), and glycerol + NaCl + sucrose + KCl at ≤0.639 water activity (−2.14 kJ g^−1^; i.e. chao‐/kosmotropicity neutral) (Fig. 7). By contrast, *X. bisporus* FRR 1522 failed to germinate on glycerol + NaCl at ≤0.688 water activity (−6.49 kJ g^−1^; i.e. mildly kosmotropic), and glycerol only at ≤0.707 water activity (18.43 kJ g^−1^ chaotropic activity); for other media its profile was the same as that of strain FRR 0025 (Fig. 7).

For media on which there was no germination of any strains, it may be that the biophysical activities of these stressor combinations/concentrations (water activity and/or chaotropicity), if also present in natural substrates, would render the latter effectively sterile and uninhabitable. Generally, germination of most strains occurred on media in the range 0.765–0.674 water activity; approximately half of strains were able to germinate from 0.668 to 0.664; and only occasional strains were able to germinate on selected media in the range 0.654–0.637 (Fig. 7a). There was no germination observed on glycerol‐only media at 0.647 or 0.635 water activity (Fig. 7), although these were beyond the known chaotropicity tolerance of these xerophiles, see below. This said, some strains are known to be, and in this study were, more chaotropicity‐tolerant than others (see below; Williams and Hallsworth, 2009). Whereas *Aspergillus* strains were generally able to germinate well on glycerol + NaCl media, salt‐containing media were relatively hostile at ≤0.651 water activity even for these strains, and there was no germination observed on any salt‐containing media at ≤0.639 water activity (Fig. 7a). In total, there were 18 media types on which no strains germinated (Fig. 7), and it is noteworthy that only two of these media were within the water‐activity range where germination took place (i.e. >0.637 water activity) (Fig. 7a).

Whereas most media which were kosmotropic (>−5.15 kJ kg^−1^) appeared hostile to fungal germination, this was attributable to their low water‐activity (i.e. <0.637) rather than kosmotropicity *per se* (Fig. 7b). One glycerol + NaCl medium in this range, however, did enable germination of *A. penicillioides* JH06THJ; water activity = 0.640 (Fig. 7b). Studies of xerophilic and halophilic microbes have not yet yielded any evidence that kosmotropic activity can act as a stress parameter which prevents microbial growth independently of osmotic stress or water activity (Williams and Hallsworth, 2009; de Lima Alves *et al*., 2015; Fox‐Powell *et al*., 2016). Several chao‐/kosmotropicity‐neutral media were hostile for germination of any strain (Fig. 7b); these were mainly glycerol + sucrose media which were <0.637 water activity (Fig. 7a). However, one chao‐/kosmotropicity neutral medium, a glycerol + NaCl + sucrose + KCl medium, was also hostile in as much as it did not permit germination of any strain (Fig. 7b); the water activity of this medium was 0.639; close to the value which prevented the germination process even for *X. bisporus* strains (Fig. 7a); its salt content, however, did not permit germination of *X. bisporus*. Glycerol‐only media with a chaotropic activity of 18.43 and 19.59 kJ kg^−1^ were hostile for germination of the majority of strains, and the glycerol‐only medium with a chaotropicity of 21.58 kJ kg^−1^ prevented germination of 11 out of the 12 strains (Fig. 7b). At 22.36 kJ kg^−1^, the next glycerol‐only medium in the range was sufficiently chaotropic to prevent germination, regardless of strain (Fig. 7b); this observation is consistent with the extreme chaotropicity of glycerol at molar concentrations (Hallsworth *et al*., 2007; Williams and Hallsworth, 2009; de Lima Alves *et al*., 2015; Stevenson *et al*., 2015a). For instance, Williams and Hallsworth (2009) reported that a medium supplemented with glycerol at 7.65 M (0.644 water activity; 20.88 kJ kg^−1^) prevented mycelial growth for a range of xerophile strains, including a number of those used in this study; a finding also consistent with the limit for *Aspergillus wentii* strain IMI 017295ii on high‐glycerol media (de Lima Alves *et al*., 2015). There was no germination on the five media with higher chaotropicity values (Fig. 7b); these media were particularly hostile because, in addition, they were below a water activity of 0.637.

### Kinetics of germination on high‐glycerol substrates

At water activities of <0.700, the shortest times for the pre‐germination phase were exhibited by *X. bisporus* FRR 0025 and FRR 3443, *X. xerophilum* FRR 0530, *A. penicillioides* JH06GBM, JH06THH and JH06THJ (between 3 and 4 days; Figs 2a, j; 4a, d, and g and h) and, for *Eurotium* spp., by *E. amstelodami* FRR 2471 and *E. echinulatum* FRR 5040 (5 days; Fig. 6a and c); and the germination process proceeded at a faster rate for *X. bisporus* and *A. penicillioides* strains than for *X. xerophilum* FRR 0530 or any *Eurotium* strain. Germ‐tube extension was most vigorous for *X. bisporus* strains FRR 2347 and FRR 3443, *X. xerophilum* FRR 0530, and *A. penicillioides* strains JH06THH and JH06THJ depending, in each case, on medium composition (Figs 2i and l; 4c, i and I); and germination occurred at <0.660 water activity only for *X. bisporus* (regardless of strain), *A. penicillioides* strains JH06GBM and JH06THJ, and *E. halophilicum* FRR 2471 (Figs 2a–l; 4d–f, j–l; 6g–i).

Collectively, these data indicate that the most‐xerophilic strains, as determined by fungal germination, were *X. bisporus* FRR 0025, *A. penicillioides* JH06GBM and JH06THJ and, for *Eurotium* species, *E. halophilicum* FRR 2471 (see also Stevenson *et al*., in press). These strains were first isolated from fruits, wooden surfaces (both JH06GBM and JH06THJ) and plant seeds respectively (Supporting Information Table S1). The germination of *X. bisporus* FRR 0025 was most rapid on glycerol + sucrose and glycerol + glucose + fructose media in the water‐activity range 0.734–0.637, according to rates of germination and germ‐tube extension (Fig. 2b and c). This preference for high‐sugar concentrations is consistent with both the primary habitats of this species (Supporting Information Table S1) and the high‐sugar preference of other *X. bisporus* strains, which generally excelled on glycerol + sucrose media (Figs 1 and 2). *A. penicillioides* JH06GBM, less particular in its required medium type, germinated vigorously on glycerol + NaCl, glycerol + sucrose, glycerol‐only and glycerol + NaCl + sucrose media in the 0.765–0.651 water‐activity range (Fig. 4d–f), a finding consistent with its ubiquity across hypersaline, high‐sugar habitats and other low water‐activity environments (Supporting Information Table S1; Samson and Lustgraaf, 1978; Arai, 2000; Zhang *et al*., 2013; Zhao *et al*., 2014; Nazareth and Gonsalves, 2014; Okano *et al*., 2015; Wei *et al*., 2015; Micheluz *et al*., 2016; Dannemiller *et al*., in press; Paulussen *et al*., in press). The progress of germination and germ‐tube extension for strain JH06GBM showed a close relation to water activity, regardless of medium composition, suggesting that this strain would make a useful model system to study water relations of xerophilic fungi (Fig. 4d–f). *Aspergillus penicillioides* JH06THJ, though unable to germinate on glycerol‐only or glycerol + glucose + fructose media, also exhibited high levels of vigour, whether germinating on glycerol + NaCl, glycerol + sucrose or glycerol + NaCl + sucrose media (Fig. 4j–I). Like *A. penicillioides*, *E. halophilicum* is somewhat indiscriminate in its habitat requirements (Supporting Information Table S1; Samson and Lustgraaf, 1978; Juarez *et al*., 2015), as evidenced by the advanced germination performance observed on glycerol + NaCl, glycerol + sucrose as well as glycerol + NaCl + sucrose media (Fig. 6g–i). For *X. bisporus*, the relatively low tolerance towards NaCl (and other salts) may arise from the lack of a Na^+^‐exporting ATPase, Ena, according to studies of its genome (Zajc *et al*., 2014; Leong *et al*., 2015). This ATPase, and alternative cation transporters, are typically present in *Aspergillus* and *Eurotium* species, and may enhance their salt‐tolerance (Miskei *et al*., 2009, Kis‐Papo *et al*., 2014).

**Figure 8:**
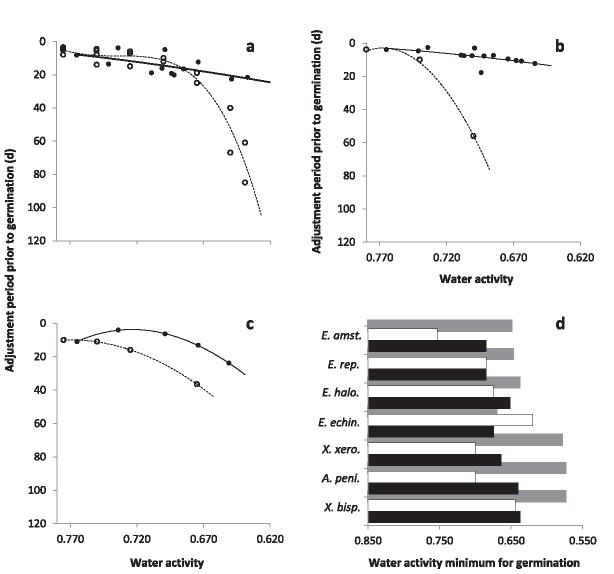
Comparisons of germination using a high‐glycerol approach (current study; solid circles/lines or solid bars) and other strategies (previous studies; open circles/dotted lines or open bars) for: *X. bisporus* FRR 1522 (**a**); *A. penicillioides* JH06GBM (current study) and FRR 3722 (Gock *et al*., 2003) (**b**); *E. halophilicum* FRR 2471 (**c**) and *X. bisporus* FRR 0025, *X. xerophilum* strains FRR 0530 (current study) and FRR 3921 (Gock *et al*., 2003), *A. penicillioides* strains JH06THJ (current study) and FRR 3722 (Gock *et al*., 2003), *E. amstelodami* FRR 2792, *E. echinulatum* FRR 5040 (current study) and an unspecified strain (Snow, 1949), *E. halophilicum* FRR 2471 and *E. repens* strains JH06JPD (current study) and FRR 2625 (Gock *et al*., 2003) (**d**). The previous studies from which data for other strains were taken are: Pitt and Hocking (1977) (**a**); Andrews and Pitt (1987) (**c**); and Pitt and Christian (1968) for *X. bisporus* FRR 0025, Wheeler and Hocking (1988) for *E. amstelodami* FRR 2792 and Andrews and Pitt (1987) for *E. halophilicum* FRR 2471 (**d**). Whereas the germination assays were carried out in current study at 30°C, all the data plotted here from all other studies came from assays carried out at 25°C. Three plots show the adjustment period prior to germination (i.e. the length of the pre‐germination phase) (**a**–**c**). Polynomial trend‐lines were constructed with Microsoft Excel; the *r* ^2^ values are 0.424 (solid line) and 0.925 (dotted line) (**a**); 0.416 (solid line) and 0.995 (dotted line) (**b**); and 0.997 (solid line) and 1 (dotted line) (**c**). The fourth plot shows the water‐activity minima for germination (**d**). For this study, black bars indicate empirical data whereas grey bars indicate theoretical minima derived by extrapolation (see *Experimental procedures*; see Stevenson *et al*., 2015a).

Of the 12 strains assayed, only four germinated on glycerol‐only media and, even for these strains, progress of germination was slow by comparison with that observed on other media (Figs 2a–c; 4a–c, d–i). A previous study which compared 42 yeast species with diverse NaCl tolerances, demonstrated a connection between degree of halotolerance or halophilicity on the one hand, and ability to take up and retain glycerol across a concentration gradient on the other (Lages *et al*., 1999). However, this factor is likely to be of lesser importance in the high‐glycerol spores and high‐glycerol substrates of this study. Diverse studies suggest that glycerol does not behave as an osmotic stressor for microbial systems and that, at molar concentrations, this solute acts as a chaotropic stressor which inhibits cellular metabolism via its ability to reduce the entropic order of membranes and/or other macromolecular systems (Hallsworth *et al*., 2003a; Chin *et al*., 2010; Cray *et al*., 2013b, 2015, 2015a; Ball and Hallsworth, 2015). Indeed, chaotropicity can become more limiting than water‐activity reduction at high concentrations of glycerol (Williams and Hallsworth, 2009; de Lima Alves *et al*., 2015). Recent work has been carried out to disentangle the biophysical constraints imposed on the xerophile *Aspergillus wentii* (i.e. chaotropicity, water‐activity, osmotic stress, ionic strength, etc) by stressors such as glycerol, sorbitol, glucose, ethanol, NaCl, KCl, MgCl_2_, NH_4_NO_3_ and urea (de Lima Alves *et al*., 2015); and those imposed on communities of halophiles by chemically diverse brines (Fox‐Powell *et al*., 2016). That biophysical constraints have considerable impacts on the kinetics of germination in the low water‐activity range (Figs 1−6 and 8) is consistent with the convergence of maximal rates for germ‐tube extension towards a common value (0.80–1 μm h^−1^) at ∼0.700 water activity, regardless of strain or species (Figs 2c, f, i and l; 4c, f, i and l; 6c, f, i and l).

Studies of microbial ecology have demonstrated that chaotropicity can determine the outcomes of competitive interactions, both qualitatively and quantitatively (Cray *et al*., 2013a, 2016, 2015). Studies of glycerol, and those of other chaotropic stressors, indicate that the biotic windows of microbes in relation to low water‐activity tolerance, and the water‐activity minima for growth, can be extended by kosmotropic substances (Hallsworth, 1998; Hallsworth *et al*., 2007; de Lima Alves *et al*., 2015; Stevenson *et al*., 2015a; Yakimov *et al*., 2015). Other data reveal that glycerol concentrations of 3.26 and 1.1 M are sufficient to maintain flexibility of cellular macromolecules, and thereby increase growth rates of xerophilic fungi and a psychrophilic yeast, at 1.7°C and −5°C respectively (Chin *et al*., 2010; C. L. Magill and J. E. Hallsworth, unpublished). Apart from the chaotropes glycerol and fructose (and glucose, which is close to chao‐/kosmotropicity‐neutral), NaCl, KCl and sucrose that were used as stressors in this study are kosmotropic (kosmotropic activities = −11.0, −11.3 and −6.92 kJ kg^−1^ M^−1^ respectively) (Cray *et al*., 2013b). For some strains, it is noteworthy that media supplemented with glycerol + kosmotropic solute(s) facilitated germination, whereas glycerol‐only media did not, and that germination only occurred at high water‐activities and/or that the process was slower on glycerol‐only media. it is for this reason that we quantified the chao‐/kosmotropic activities of the media, which ranged from a kosmotropic activity of −24.90 kJ kg^−1^ M^−1^ for the 0.575 water‐activity glycerol + NaCl medium to a chaotropic activity of 29.05 kJ kg^−1^ M^−1^ for the 0.585 water‐activity glycerol‐only medium (Supporting Information Table S2).

The water‐activity range assayed in this study was considered in the context of the entire windows for germination of *A. penicillioides* JH06THJ, *E. halophilicum* FRR 2471 and *X. bisporus* FRR 0025 on their preferred media (glycerol + NaCl, or glycerol + NaCl + sucrose, and glycerol + sucrose respectively) (Stevenson *et al*., in press). The germination rates in the water‐activity range 0.765–0.637 were between 37.5% and 0.02% of the optimum rates (typically observed close to 0.900 water activity), depending on the strain and culture‐medium (Stevenson *et al*., in press). Similarly, pH and temperature curves for these three strains (Stevenson *et al*., in press) confirm that the pH of media used in this study (5.3–7.0; Supporting Information Table S2) were within the optimal pH range (typically 4.5–7.5), and that the 30°C incubation‐temperature used in this study was optimal regardless of strain.

For each species of xerophile assayed, the kinetics of germination reported in the current study were more rapid than those reported previously. Below 0.700 water activity, germination had been observed only after a lag period of 38–80 days prior to this study, regardless of species (Pitt and Christian, 1968; Pitt and Hocking, 1977; Andrews and Pitt, 1987). The discrepancy between high‐glycerol germination (current study) and the findings of earlier studies was more apparent at lower water‐activity values, and was most prominent at ≤0.700 water activity. For instance, germination of *A. penicillioides*, *E. halophilicum* and *X. bisporus* in the high‐glycerol system occurred 16–73 days earlier in the water‐activity range 0.700–0.640, depending on fungal species (Fig. 8a–c). Furthermore, (with the exception of *E. echinulatum*), germination took place at lower water activities in the current study, regardless of species (Fig. 8d). Germination of *X. bisporus* FRR 0025 ascospores occurred at 0.637 water activity after 18.7 days (Fig. 2a), surpassing the previously established limit of 0.644 after 80 days (Fig. 8a) at a germ‐tube growth‐rate of 0.0104 mm d^−1^ which is equivalent to 3.80 mm year^−1^. This germ‐tube extension rate is consistent with hyphal growth rates of 13.14 and 5.48 mm year^−1^ reported for *X. bisporus* strains FRR 2347 and FRR 3443, respectively, on high‐glycerol media at the slightly higher water activity of 0.640 (Stevenson *et al*., 2015a). It should be noted, however, that earlier studies were carried out at 25°C (see Fig. 8), and we now know that germination of these xerophiles is usually optimal at 30°C (Stevenson *et al*., in press) so it may be that part of the differences observed in Fig. 2 also relate to this temperature difference.

High levels of intracellular glycerol are known to increase rates of germination and germ‐tube growth at moderate water‐activity values (and, indeed, at high concentrations of ethanol), for non‐xerophilic fungi (Hallsworth and Magan, 1994a, 1995; Hallsworth *et al*., 2003b). Furthermore, high‐glycerol spores of non‐xerophiles, such as *Metarhizium anisopliae* and *Paecilomyces farinosus*, were able to germinate and develop germ tubes at lower water‐activities (≤0.887 and 0.923, respectively) than low‐glycerol spores; i.e. ≥0.989 (Hallsworth and Magan, 1995). This information is consistent with the findings of this study, and the key roles played by glycerol under conditions which impose biophysical stresses. Recent analyses of microbial growth kinetics suggest that cellular systems are sensitive to differences of ±0.001 water activity (Stevenson *et al*., 2015b). The curves for germination and germ‐tube growth of *X. bisporus* FRR 2347 (Fig. 2h and i) and *A. penicillioides* JH06THH (Fig. 4h and i), respectively, which indicate sharp decreases in the progress of germination for each 0.002 decrease of water activity, are consistent with this finding.

### Extrapolations suggest water‐activity minima of <0.600 for fungal germination

According to empirical determinations, the water‐activity minima for spore germination of the most xerophilic strains were: 0.637 for *X. bisporus* on glycerol + sucrose supplemented media (all four strains tested), 0.640 for *A. penicillioides* strain JH06THJ on glycerol + NaCl‐supplemented media, and 0.651 for *E. halophilicum* strain FRR 2471 and *A. penicillioides* strains JH06GBM and JH06THJ on glycerol + NaCl + sucrose‐supplemented media (Figs 2; 4e, f, k and l; 6h and i). All spores that germinated in this study went on to form mycelium which covered the surface of the medium (data not shown). The water‐activity limits reported here are comparable with Snow's (1949) 0.640‐water activity limit for germination. However, the assessment period used in this study was relatively short (50 days, rather than 2 years); despite this, germination was extremely rapid (Fig. 8) and trends for the progress of germination of five strains indicate theoretical water‐activity minima of <0.600 (see below). Furthermore, the removal of Petri‐plate lids resulted in a reduction of culture‐medium water activity of up to 0.003 during the course of the experiment (data not shown), and might have thereby prevented germination on media of less than 0.637 water activity (Figs 1 and 2).

For studies of solute stress in microbial cells, especially those carried out at <0.755 water activity, stressor solubility can restrict the types of experimental approach which can be employed to determine water‐activity minima (Stevenson *et al*., 2015a). However, theoretical water‐activity minima can be derived via extrapolation of datasets for planktonic growth‐, hyphal extension‐ and germination‐rates and have been experimentally validated (Rosso and Robinson, 2001; Tassou *et al*., 2007; Huchet *et al*., 2013; Stevenson *et al*., 2015a). In the current study, theoretically determined water‐activity minima were derived by extrapolation of trend lines for strains which germinated at ≤0.674 water activity for three or more water‐activity values on the same type of medium, or two data points with the lower water‐activity value at <0.665 (Figs 2b, c, e, f, h, i, k and l; 4b, c, e, f, h, i, k and l; and 6e, f, h and i). These water‐activity minima were: <0.570 for *A. penicillioides* strains JH06GBM and JH06THH on glycerol‐only media (Fig. 4e, f, h and i), and *X. bisporus* strain FRR 0025 on glycerol‐only and glycerol + sucrose media (Fig. 2b and c); <0.575 for *X. xerophilum* strain FRR 0530 on glycerol + NaCl and glycerol+sucrose media (Fig. 4b and c); <0.600 for *X. bisporus* strains FRR 0025 and FRR 1522 on glycerol + glucose + fructose media (Fig. 2b, c, e and f); 0.646 (glycerol + NaCl) and 0.635 (glycerol + NaCl + sucrose) for *E. halophilicum* (Fig. 6h and i); and 0.655 for *E. echinulatum* FRR 5040 on glycerol + sucrose media (Fig. 6e and f).

During the 50‐day assessment period, none of these strains germinated below the empirically determined water‐activity limits reported above (Supporting Information Table S2; Fig. 7). However, a prolonged lag‐phase is typical even for extreme xerophiles at water‐activity values of <0.640 (i.e. 4 months to 2 or more years, according to Snow [1949] and Pitt and Christian [1968]). Furthermore, there is a disconnect between the length of the pre‐germination phase and subsequent rates of germination and germ‐tube extension. For instance, germination and germ‐tube extension rates were comparable at 0.765 and 0.649 water activity (on glycerol + NaCl and glycerol + glucose + fructose media, respectively) for *X. bisporus* strain FRR 0025 (Figs 1a and b; 2a–c); at 0.709 and 0.694 water activity (on glycerol + NaCl and glycerol + glucose + fructose media, respectively) for *X. xerophilum* FRR 0530 (Figs 3a and b; 4a–c); and at 0.699 and 0.694 water activity (on glycerol + sucrose and glycerol + glucose + fructose media, respectively) for *A. penicillioides* strain JH06GBM (Figs 3c and d; 4d–f), and yet in each case there was a 15‐day time interval between the commencement of germination on the different types of medium. It is plausible, therefore, that germination could occur in high‐glycerol systems at <0.637 water activity or over timescales of >50 days: it has already been established that detectable growth of microbes, if not single cell divisions, in diverse types of habitats can take place over periods of years or decades (see Johnston and Vestal, 1991; Sun and Friedmann, 1999; Parkes *et al*., 2000; D'Hondt *et al*., 2002; Lomstein *et al*., 2012). Previous studies have demonstrated the fluidity of microbial growth windows in relation to biophysical parameters. For instance, chaotropes can reduce temperature minima for specific microbes by 5–10°C (Chin *et al*., 2010; Cray *et al*., 2015a); a phenomenon which has also been observed for fungi growing at low temperature on high‐glycerol media (see above). It may be, therefore, that fungi could germinate on the hostile media listed above under environmental conditions other than those used in the current study.

### Additional implications for microbial ecology

In the experimental system used in this study, *A. penicillioides* was vigorous (Figs 3c and d; 4d–f) relative to the slower‐growing *X. bisporus* (Figs 1 and 2); the latter is a specialist fungus which has a low competitive ability (Leong *et al*., 2015). However, like the proverbial hare and tortoise, it is the slower of these two—*X. bisporus*—which ultimately germinates at the lowest water activity (Fig. 2b, c, e, f, h, i, k and l). For instance, *A. penicillioides* JH06GBM had germinated, or was germinating, on nine types of media by Day 10, and all germination was complete by Day 22, regardless of water activity (3c and 4d), whereas *X. bisporus* FRR 2347 was germinating on only three types of media by Day 10, and all germination was complete by Day 35 (Figs 1e and 2g). However, *X. bisporus* FRR 2347 germinated down to 0.649 and 0.637 on glycerol + glucose + fructose and glycerol + sucrose media, respectively, whereas *A. penicillioides* JH06GBM did not germinate on any media below 0.651 water activity (Figs 2i and 4f).

This study, like other recent studies, has confirmed that water activity acts as a universal life‐limiting parameter. For instance, there is a convergence of water‐activity minima towards a common value for: extremophiles of each domain of life (see below); spores and hyphae of fungi such as *X. bisporus* (see above); and diverse fungal xerophiles such as *X. bisporus* and *A. penicillioides* (see theoretical minima for germination in Figs 2 and 4, and mycelial limits in Stevenson *et al*., 2015a). This said, other parameters can influence the water‐activity windows for microbial activity: chaotropicity, turgor changes, ionic strength, temperature, pH, nutritional factors, etc (Williams and Hallsworth, 2009; de Lima Alves *et al*., 2015; Harrison *et al*., 2015; Stevenson *et al*., 2015a; Fox‐Powell *et al*., 2016). Based on manipulations of these interacting parameters, recent studies have revealed that extremely halophilic bacteria and Archaea are not constrained to water activities of ≥0.755 (equivalent to saturated NaCl), but retain activity down to values close to 0.600 water activity (see above); and the lower limit for mycelial growth of extremely xerophilic fungi has been revised from 0.656 (Pitt and Christian, 1968) to 0.640 water activity, with a theoretical minimum of 0.632 (Stevenson *et al*., 2015a). The findings of this study suggest that metabolism and multiplication of some microbes is plausible at <0.605 water activity, and it may be that intra‐ and/or extracellular glycerol can facilitate this in some natural habitats of fungal xerophiles. Indeed, there is evidence to suggest that fungal spores produced in nature on low water‐activity substrates selectively accumulate low molecular weight polyols such as erythritol or glycerol; see also below.

Whereas there have been studies of glycerol production and utilization for natural microbial communities *in situ* (e.g. Oren, in press), little is known about the biophysics of glycerol in relation to ecosystem function. For instance, glycerol is hygroscopic in nature and so may draw external water into microbial biomass that is located in water‐constrained habitats; applications of glycerol can correct water‐repellency in non‐wetting sandy soils (Bonnardeaux, 2006) and increase soil organic‐carbon content (Qian *et al*., 2011). It may be, therefore, that knowledge‐based approaches to manipulate the microbial ecology of glycerol can be used to enhance ecosystem development (e.g. to enhance function of soil saprotrophs or encourage desirable plant:microbe interactions) in arid environments. We do know that mesophilic microbes, as well as xerophile systems, can depend on glycerol for optimal function, e.g. Hallsworth and Magan (1995) and Mattenberger *et al*. (in press). The high‐glycerol system used in this study can be viewed as an anthropogenic intervention in the usual biology of the fungal system. However, both artificial and natural fungal substrates which have stressfully low water‐activity values yield spores with high amounts of the low‐*M*_r_ polyols glycerol and, to a lesser extent, erythritol (Hallsworth and Magan, 1994a,b,c; 1995; Magan, 2001; Hallsworth *et al*., 2003a; Andersen *et al*., 2006; Bhaganna *et al*., 2010; Rangel *et al*., 2015a). This includes, for instance, conidia produced by entomopathogenic fungi on the insect cadaver (Magan, 2001). Furthermore, microbial cells can efficiently sequester trace amounts of glycerol present in their environment according to studies of halophilic species (Oren and Gurevich, 1994; Oren, 1995, 2010), and studies of both mesophilic and xerophilic fungi demonstrate that glycerol can be taken up and retained in the cytosol under hyperosmotic conditions (Hallsworth and Magan, 1994c; de Lima Alves *et al*., 2015). The findings of this study are, therefore, pertinent to fungi in natural habitats. Further, a recent study of an extreme halophilic archaeon, *Natrinema pallidum*, demonstrated that, when present in brines, glycerol helped to reduce the water‐activity minima for growth to an unprecedented value; 0.681 (Stevenson *et al*., 2015a). Glycerol can also boost the stress tolerance of bacterial cells (Vilhelmsson and Miller, 2002; Bhaganna *et al*., 2010; 2016). It is, therefore, plausible if not, indeed, likely that glycerol facilitates the activity of diverse types of microbial cell/community under low water‐activity conditions. In this way, glycerol may determine the extent of, and failure points for, the functional biosphere on Earth (Hallsworth *et al*., 2007; Stevenson *et al*., 2015b; Yakimov *et al*., 2015). This has implications in the field of astrobiology, as glycerol can enhance macromolecular flexibility at low temperature and may also facilitate the habitability of brines which are found on other extraterrestrial bodies.

### Conclusions

The findings presented in this study indicate that, whereas some xerophiles have requirements for additional solutes, glycerol catalyzes spore germination at the water activities corresponding to the limits for microbial life. The combination of high concentrations of intra‐ and extracellular glycerol can enhance both the kinetics of germination at water activities down to 0.637, and reduce the water‐activity minima for biotic activity of individual strains. The only undisputed reports for microbial growth or germination below the 0.637 water‐activity limit for conidial germination of *X. bisporus* (current study) are those of halophilic Archaea growing in bittern brine (at 0.644; Javor, 1984; Stevenson *et al*., 2015a), and *X. bisporus* aleuriospores germinating at 0.605 (Pitt and Christian, 1968) (for discussions of the various controversial and unsubstantiated reports, see Pitt and Christian, 1968; Hallsworth *et al*., 2007; Stevenson and Hallsworth, 2014; Stevenson *et al*., 2015b). Whereas these findings have yet to be reproduced, we have no reason to doubt these reports. Indeed, the promotion of xerophile germination by glycerol (Figs 1−6 and 8) suggests that microbial activity can occur at ≤0.600 water activity. The relatively high germination rates observed for some strains in the range 0.654–0.637 water activity and the low theoretical water‐activity minima, determined via extrapolation of data ‐ i.e. down to 0.570‐ (Figs 2, 4 and 6), act as strong indicators that extreme xerophiles are capable of metabolic activity and structural growth under hitherto unprecedented conditions.

Understanding the biophysical and ecophysiological factors which interact to enable and constrain life has important implications. The majority of microbiological studies, even for extremophilic taxa, focus on organisms growing under relatively benign conditions. Characterizing life under hostile conditions may be critical to understand and manipulate nutrient cycling in the biosphere; saprotrophic activity in arid soils for instance. Furthermore, understanding what is (and is not) possible on Earth will inform our search for potential habitats on other planets. Some infectious organisms can inhabit low water‐activity environments (either in the human body or in spaces cohabited with humans) and so knowledge of these limits may facilitate novel antimicrobial treatments or sterilisation techniques based on reducing the water availability below these limits. Many microbe‐driven industrial processes, including food‐, drinks‐ and biofuel fermentations take place in low water‐activity milieu (Cray *et al*., 2015a). Greater understanding of fungal physiology, and fungal interactions with other microbes (e.g. Cray *et al*., 2013a; 2015b; Rangel *et al*., 2015b), will facilitate their greater exploitation in biotechnology (in a similar way to the use of thermophiles and their heat‐tolerant biomolecules). Furthermore, our work here suggests that careful supplementation of fermentation media with glycerol (possibly in combination with other stressors) can enable the rational manipulation of microbial metabolism and/or cell division targeted towards specific industrial applications.

The findings of this study represent a paradox. On the one hand, glycerol can be exceptionally stressful and prevent cellular development. On the other hand, this simple polyol may be essential for cells to function at the water‐activity limit for life, and this raises further intriguing questions. Several studies have shown that the DNA of metabolically active cells becomes disordered/damaged below 0.600 (Falk *et al*., 1963; Asada *et al*., 1979); is it possible that glycerol can mitigate against this failure? What other component(s) of the cell or its metabolism fail(s) at low water‐activity; e.g. the cell membrane or interactions between macromolecular systems; or is there a prohibitive energy requirement at <0.600 water activity as suggested by Hocking (1993)? Glycerol is infinitely soluble, and highly effective in water‐activity reduction, has diverse roles in cellular stress‐protection, can expand both vigour and windows for biotic activity in the context of mechanistically diverse sources of stress, and can enable growth and/or preserve cellular structures at sub‐zero temperatures; and yet can ultimately act as a stressor itself. It is nevertheless certain that the biophysical activities of glycerol intervene in interactions between solutes, macromolecular systems, and/or water. Further work is needed to see whether the glycerol in foods (included that which is added as a humectant) may inadvertently enhance food spoilage by promoting microbial activity at low temperature and/or low water activity. Glycerol is the key molecule used by microbes to mitigate a variety of stressful conditions, and this study demonstrated that glycerol enables microbial metabolism beyond the usual water‐activity constraints. There are special substances in biology and biochemistry; nothing acts in heredity like DNA; phosphate is unique in its activity as a buffer; water is effectively irreplaceable as the milieu for life, as is carbon as a versatile bonding atom or oxygen as a terminal oxidising agent. And likewise, while other compatible solutes are undoubtedly important, it is glycerol that represents the most‐ideal and most‐special stress‐protectant in many circumstances. The exact mechanisms by which water‐activity curtails cellular function on the one hand, and glycerol can mitigates this on the other, nevertheless remain enigmatic.

## Experimental procedures

### Fungal isolates and culture conditions

*Aspergillus penicillioides* strains JH06GBM, JH06THJ and JH06THH, and *E. repens* strain JH06JPD were isolated by Williams and Hallsworth (2009) and are available from the corresponding author of this article. *E. amstelodami* strain FRR 2792, *E. echinulatum* strain FRR 5040, *E. halophilicum* strain FRR 2471, *X. xerophilum* strain FRR 0530, and *X. bisporus* strains FRR 0025, FRR 1522, FRR 2347 and FRR 3443 were obtained from CSIRO Food and Nutritional Sciences Culture Collection (North Ryde, NSW, Australia). Please note that *E. echinulatum*, *E. halophilicum* and *E. repens* have recently been renamed as *Aspergillus brunneus*, *Aspergillus halophilicus* and *Aspergillus pseudoglaucus* respectively (Hubka *et al*., 2013). Additional information on each xerophile strain is given in Supporting Information Table S1. Cultures were maintained on Malt Extract Yeast Extract Phosphate Agar (MYPiA; 10 g malt extract, 10 g yeast extract, 1 g anhydrous K_2_HPO_4_, agar 15 g l^−1^) supplemented with 5.5 M glycerol (0.821 water activity) at 30°C.

Only 13 microbial species/communities have been observed to grow and/or germinate in the water‐activity range 0.690–0.605 according to empirical data obtained from experiments carried out *in vitro* or, for microbial habitats, *in situ* (Williams and Hallsworth, 2009; Stevenson *et al*., 2015a, 2015b). The majority of these are fungal xerophiles, and many of the strains used in the current study were amongst them: *X. bisporus* FRR 0025 (the strain reported by Pitt and Christian [1968] to have 0.644‐ and 0.605‐water activity limits for germination of ascospores and aleuriospores respectively), FRR 1522, FRR 2347 and FRR 3443; *X. xerophilum* FRR 0530; *A. penicillioides* JH06GBM, JH06THJ and JH06THH; *E. amstelodami* FRR 2792; *E. echinulatum* FRR 5040; *E. halophilicum* FRR 2471 and *E. repens* JH06JPD. Such xerophiles are commonly found in high‐glycerol and/or sugar‐rich habitats (Pitt, 1975; Lievens *et al*., 2015). However, *A. penicillioides* and *Eurotium* spp. are also found in other environments, such as solar salterns, crystallizer ponds and house dust (see above, and Supporting Information Table S1); *A. penicillioides*, *Eurotium* spp., *X. bisporus* (e.g. strain CBS 328.83) and *X. xerophilum* are found within saprotroph communities on surfaces such as dried leaves, straw, wood and paper (Supporting Information Table S1; Arai, 2000; Wang *et al*., 2001; Williams and Hallsworth, 2009; Cray *et al*., 2013a; Juarez *et al*., 2015).

### Production of spores and quantitation of glycerol content

Each xerophile strain was cultured on MYPiA supplemented with glycerol (5.5 M; 0.821 water activity) for 10–14 days for *A. penicillioides*, *E. amstelodami*, *E. echinulatum* and *E. repens*; and for 21–28 days for the slower‐growing/later‐sporulating *E. halophilicum*, *X. bisporus* and *X. xerophilum* at 30°C. Media were inoculated using 2‐mm‐diameter plugs of agar taken from the periphery of an exponential‐phase culture growing on medium of the same composition, and plates of each medium were placed in a sealed bag of low‐density polyethylene to maintain a constant relative humidity (thus maintaining water activity), while allowing gaseous exchange (Hallsworth *et al*., 1998). Spores were harvested into a sterile solution of NaCl (5.3 M) and the resulting suspensions were filtered through glass wool to remove hyphal fragments. Spore samples were then freeze‐dried and extractions were carried out with 5‐mg samples of spores that were sonicated in AnalaR water (Merck, Darmstadt, Germany) and then immersed in a boiling water bath as described previously (Hallsworth and Magan, 1997). Samples were sonicated for 120 s and placed into a boiling water bath for 5.5 min as described by (Hallsworth and Magan, 1997). Extracts were filtered (through a 0.2‐μm filter) and then injected onto a ICS‐3000 Dionex Ion Chromatography System (Dionex, Sunnyvale, CA, USA) fitted with a CarboPac MA1 plus guard column (Dionex), and they were quantified by pulsed electrochemical detection based on the protocol reported in Hallsworth and Magan (1997). The mobile phase was 100 mM NaOH (pH 14), and the flow rate was 1 ml min^−1^ and the limits of detection were 1.6 µg ml^−1^ glycerol.

### Characterization of germination of high‐glycerol propagules at low water‐activity on media supplemented with diverse stressors

For the 12 xerophile strains, ability to germinate and germination kinetics at biologically hostile water activities (0.765–0.570) were assessed using a range of 36 media (Supporting Information Table S2), designed to emulate physicochemical stresses experienced by microbes in both natural habitats and anthropogenic systems; these media include MYPiA supplemented with glycerol + NaCl; glycerol + sucrose; glycerol + glucose + fructose; glycerol only; glycerol + NaCl + sucrose and glycerol + NaCl + KCl + sucrose (see above). All media used in this study were incubated in polyethylene bags with identical media types at 30°C (except for those used in temperature assays described above), and were sterilized by autoclaving at 121°C (1 atm) except for those containing glucose + fructose.^3^ These were maintained in a water bath set at 80°C for 30 min to avoid reactions that would lead to the production of inhibitory substances. Spores were harvested (see above), inoculated (see below) onto the media listed in Supporting Information Table S2, and germination was assessed, over a period of 50 days, as described below.

Data from germination assays were used to plot percentage germination and germ‐tube length versus time (Figs 1, 3 and 5). Plots of percentage germination versus time were used to determine length of the pre‐germination phase (i.e. the adjustment period prior to germination), by extrapolating fitted polynomial trend lines to a point of 0% spore germination for each media type (data not shown). These plots were also used to determine maximum rate of progress of germination; i.e. during the exponential phase (data not shown). Similarly, the plots of germ‐tube length were used to determine maximum rate of germ‐tube development; i.e. during the exponential phase (data not shown). Data for the length of the pre‐germination phase, and maximum rates of progress of germination and germ‐tube development were then plotted versus water activity (Figs 2, 4 and 6).

### Assessment of germination; comparison with previous studies

Spores were obtained from cultures incubated on MYPiA + glycerol (5.5 M) for 10–14 days for strains of *A. penicillioides*, *E. amstelodami*, *E. echinulatum* and *E. repens*; and 21–28 days for strains of *X. bisporus, X. xerophilum* and *E. halophilicum*; *X. bisporus, X. xerophilum and E. halophilicum*. Spores were harvested from colonies growing on MYPiA + glycerol (5.5 M) media by covering Petri plates with sterile solutions of 5.5 M glycerol (15 ml); aerial spores were then dislodged by gently brushing with a sterile glass rod. The resulting suspension was then passed through sterile glass wool twice to remove hyphal fragments as described in earlier studies (Hallsworth and Magan, 1995; Chin *et al*., 2010). Spore suspensions were then adjusted to a final spore concentration of 1 × 10^6^ spores ml^−1^. Inoculation of media was carried out by pipetting spore suspension (150 ųl) onto the medium; the suspension was then distributed across the agar surface using a sterilize glass spreader.

Germination was assessed by removing a 4‐mm agar disc, and immediately quantifying percentage germination, spore diameter and germ‐tube length using light microscope. Plates were immediately resealed and placed back in the incubator after removal of agar discs. Percentage germination was determined via counts of 200 spores, and 50 individual germinated spores were measured for germ‐tube length; spores with germ‐tubes longer than their diameter were considered to have germinated (Hallsworth and Magan, 1995). In each case, percentage germination and mean germ‐tube length were determined for isolated spores and were not assessed for any spores located in clumps. Assessments were made at least daily over a 50‐day period and all measurements were carried out in triplicate.

Data obtained from low water‐activity germination assays (in the range 0.765–0.570) were presented as percentage spore germination over time (Figs 1a, c, e and g; 3a, c, e and g; 5a, c, e and g), and germ‐tube length over time (Figs 1b, d, f and h; 3b, d, f and h; 5b, d, f and h). The length of pre‐germination phase (Figs 2a, d, g and j; 4a, d, g and j; 6a, d, g and j) was determined by extrapolation of percentage spore‐germination plots (Figs 1a, c, e and g; 3a, c, e and g; 5a, c, e and 5g), which was carried out using polynomial regression analysis as described below (data not shown), and then plotted against water activity. Plots for maximum rate of progress of germination versus water activity (Figs 2b, e, h and k; 4b, e, h and k; 6b, e, h and k) and maximum rate of germ‐tube development versus water activity (Figs 2c, f, i and l; 4c, f, i and l; 6c, f, i and 6l) were constructed by determining exponential rates for germination (% of total) and germ‐tube length against time as plotted in Figs 1, 3 and 5.

Germination kinetics for the three model strains, which had been cultured on high‐glycerol media, were compared with those from extant datasets (Fig. 8). For *X. bisporus* strain FRR 1522, the pre‐germination phase for high‐glycerol spores (for water‐activity range 0.780–0.620; current study) were plotted against those for spores harvested from a basal medium containing 2% w/v glucose (Pitt and Hocking, 1977; Fig. 8a). Times for the inception of germination for high‐glycerol spores of *A. penicillioides* strain JH06GBM (for water‐activity range 0.780–0.620; current study) were plotted against the highest rates available for *A. penicillioides* strain FRR 3722, which had been pre‐cultured on a basal medium with no added solutes (Gock *et al*., 2003; Fig. 8b). For *E. halophilicum* strain FRR 2792, the most‐rapid germination data for high‐glycerol spores at a given water activity were plotted against those for spores obtained from a basal medium containing 2% w/v glucose (Andrews and Pitt, 1987; Fig. 8c). Polynomial regression was applied to each dataset, utilising the highest regression coefficient, as described by Stevenson *et al*. (2015a), and the lowest water activities at which germination was observed in the current study are summarised and compared with those reported for each of the extant datasets (Fig. 8d). Data were obtained from: Pitt and Christian (1968; for ascospores of *X. bisporus*); Gock *et al*. (2003; for *A. penicillioides, X. xerophilum* and *E. repens*); Snow (1949; for *E. echinulatum*); Andrews and Pitt (1987; for *E. halophilicum*) and Wheeler and Hocking (1988; for *E. amstelodami*) for use in Fig. 8d. Theoretical water‐activity minima for the fungal strains used in this study were determined by extrapolating linear trend‐lines of maximum rates of germination versus water activity (Figs 2b; 4e; 6b, e, h and k).

### Quantitation of culture‐medium water activity, chao‐/kosmotropicity and pH

The water activity of all media was determined empirically using a Novasina Humidat‐IC‐II water‐activity machine fitted with an alcohol‐resistant humidity sensor and eVALC alcohol filter (Novasina, Pfäffikon, Switzerland). Water‐activity measurements were taken at the same temperature at which cultures and germination assays were to be incubated, and several precautions were employed to ensure accuracy of readings, as described previously (Hallsworth and Nomura, 1999; Stevenson *et al*., 2015a). The instrument was calibrated between each measurement using saturated salt solutions of known water activity (Winston and Bates, 1960). The water activity of each medium type was determined three times, and variation was within ±0.001. Media chao‐/kosmotropicity values were determined using the agar‐gelation method described by Cray *et al*. (2013b). Extra‐pure reagent‐grade agar (Nacalai Tesque, Kyoto, Japan), at 1.5% w/v and supplemented with stressors at the concentrations used in the medium, was used to determine chao‐/kosmotropicity values for added solutes (see Hallsworth *et al*., 2003b; Cray *et al*., 2013b). A Cecil E2501 spectrophotometer fitted with a thermoelectrically controlled heating block was used to determine the wavelength and absorbance values at which to assay compounds, and values for chao‐/kosmotropic activity were calculated relative to those of the control (no added solute) as described by Cray *et al*. (2013b). The pH values for pre‐autoclaved media were determined using a Mettler Toledo Seven Easy pH‐probe (Mettler Toledo, Greifensee, Switzerland); values for solid media (post‐autoclaved) were determined prior to inoculation using Fisherbrand colour‐fixed pH indicator sticks (Fisher Scientific Ltd, Leicestershire, UK).

## Supporting information

Additional Supporting Information may be found in the online version of this article at the publisher's web‐site:

**Table S1.** Overview of the 12 xerophile strains, all in the Aspergillaceae lineage of the Eurotiales, that were used in the current study.
**Table S2.** Media used for germination assays
**Table S2.** Media used for germination assays
Supplementary figure legends **Figure S1.** Glycerol content of spores of each xerophile strain that had been cultured on MYPiA supplemented with glycerol (5.5 M; 0.821 water activity) at 30°C. Data are means of three replicates, and grey bars indicate standard errors.
**Supporting references**

## Footnotes

1 These factors are not always mutually exclusive (Hallsworth, 1997; Hallsworth *et al*., 2015; de Lima Alves *et al*., 2015).

2 A number of reports show that, at intermediate water‐activity values, arabitol and/or erythritol are the primary polyols which accumulate (Hallsworth and Magan, 1994b, c; 1995; Andersen *et al*, 1987; Rangel *et al*., 2014).

3 A post‐autoclave analysis of media containing sucrose was not carried out, though there is a theoretical possibility that some of the sucrose hydrolyzed to form glucose and fructose.
