# Supplementary material for: Glycerol enhances fungal germination at the water‐activity limit for life
Source: Environ Microbiol. 2016 Nov 13;19(3):947–67. doi: 10.1111/1462-2920.13530 (PMC5363249; doi:10.1111/1462-2920.13530)
Supplement: Supplementary file 1 — Table S1. Overview of the 12 xerophile strains, all in the Aspergillaceae lineage of the Eurotiales, that were used in the current study. Table S2. Media used for germination assays Table S2. Media used for germination assays Supplementary figure legends Figure S1. Glycerol content of spores of each xerophile strain that had been cultured on MYPiA supplemented with glycerol (5.5 M; 0.821 water activity) at 30°C. Data are means of three replicates, and grey bars indicate standard errors. Supporting references [file EMI-19-947-s001.docx]

**Supporting information**

**Table of contents:**

**Supplementary tables**

**Table S1.** Overview of the 12 xerophile strains, all in the Aspergillaceae lineage of the Eurotiales, that were used in the current study.

**Table S2**. Media used for germination assays

**Supplementary figure legends**

**Figure S1**. Glycerol content of spores of each xerophile strain that had been cultured on MYPiA supplemented with glycerol (5.5 M; 0.821 water activity) at 30°C. Data are means of three replicates, and grey bars indicate standard errors.

**Supporting references**

**Table S1.** Overview of the 12 xerophile strains, all in the Aspergillaceae lineage of the Eurotiales, that were used in the current study.

**Species and strain Substrate of isola- Accession Studies of stress biology, taxonomy and ecology**

**designation tion (country and numbers for**

**date of isolation) other culture**

**collections**^a^

___________________________________________________________________________________________________________________

***Strains within the Monascus* : *Xeromyces* : *Leiothecium* clade**

*Xeromyces bisporus*  High-moisture ATCC 28298 Studies of xerophilicity have demonstrated germination of ascospores and

FRR 0025 prunes (Australia, aleuriospores at 0.644 and 0.605 water activity, respectively (Pitt and Christian,

1968) 1968), and mycelial extension down to 0.656 (Williams and Hallsworth, 2009) and

0.640 water activity, on media supplemented with glycerol+glucose+fructose and glycerol+sucrose+NaCl+KCl, respectively (Stevenson *et al*., 2015a). Water : temperature relations have been characterized on glycerol-supplemented media (Williams and Hallsworth, 2009). Included in studies to explore the phylogenetic distinction between the *Monascus* and *Xeromyces* species (Park and Jong, 2003).

*Xeromyces bisporus* Spoiled liquorice ATCC 3694 A study of xerophilicity demonstrated mycelial extension down to 0.653 water activ-

FRR 1522 (Australia, 1973) IMI 317904 ity, on media supplemented with 7.60 M glycerol (Williams and Hallsworth, 2009).

Water : temperature relations have been characterized on glycerol-, NaCl- and glucose+fructose and glycerol-supplemented media (Pitt and Hocking, 1977; Gock *et al*., 2003). This strain was included in the phylogenetic characterization of the genera *Monascus* and *Xeromyces*; analysis of culture extracts failed to detect secondary metabolites (Park and Jong, 2003; Petterson *et al*., 2011; Leong *et al*., 2015).

*Xeromyces bisporus* Mouldy fruit cake IMI 317903 Studies of xerophilicity have demonstrated mycelial extension down to 0.653 and

FRR 2347 (Australia, 1980) 0.640 water activity, on media supplemented with 7.60 M glycerol and glycerol+suc-

rose+NaCl+KCl, respectively (Williams and Hallsworth, 2009; Stevenson *et al*., 2015a). Water : temperature relations have been characterized on glycerol-, and glucose+fructose-supplemented media (Gock et al., 2003; Williams and Hallsworth, 2009; Leong *et al.* 2011). Conidia tenacity of this strain was studied to determine interactions between the chao- or kosmotropic solutes and exposure to extreme temperatures and pressure (Chin *et al*., 2010). Ability to compete with other xerophilic species at ≤ 0.800 water activity results from the relatively rapid growth of this *X. bisporus* strain (Leong *et al*., 2011)*.* This strain, was included in the phylogenetic characterization of the genera *Monascus* and *Xeromyces*; analysis of culture extracts failed to detect secondary metabolites (Petterson *et al*., 2011; Leong *et al*., 2015).

*Xeromyces bisporus* Raisins (Australia, IMI 317901 Studies of xerophilicity have demonstrated mycelial extension down to 0.653 and

FRR 3443 1967) 0.640 water activity, on media supplemented with 7.60 M glycerol and glycerol+suc-

rose+NaCl+KCl, respectively (Williams and Hallsworth, 2009; Stevenson *et al*., 2015a). Water : temperature relations have been characterized on glycerol-supplemented media (Williams and Hallsworth, 2009). This strain, was included in the phylogenetic characterization of the genera *Monascus* and *Xeromyces*; analysis of culture extracts failed to detect secondary metabolites (Petterson *et al*., 2011; Leong *et al*., 2015).

*Xerochrysium xerophilum* High-moisture ATCC 18052 Studies of xerophilicity have demonstrated germination of aleuriospores at 0.708

(formerly *Chrysosporium* prunes (Australia, CBS 153.67 water activity (Pitt and Christian, 1968), and mycelial extension down to 0.686 (Wil-

*xerophilum*) FRR 0530^T^ 1962) IMI 126287 liams and Hallsworth, 2009) and 0.660 water activity (Leong *et al*., 2011), on media

UAMH 2368 supplemented with glycerol and glucose+fructose, respectively. Water : temperature

relations have been characterized on glucose+fructose-supplemented media (Leong *et al*., 2011). This strain was used in studies showing growth at increased carbon dioxide and reduced oxygen levels (Kinderlerer, 1997), facilitated by fermentative metabolism (Kinderlerer, 1987). FRR 0530^T^ is the Type strain of *X. xerophilum* which was formerly *Chrysosporium xerophilum* (Pitt, 1966; Pitt *et al*., 2013), and is the closest known relative to *X. bisporus* (Petterson *et al*., 2011). Inhibits growth of *X. bisporus*, possibly via the production of volatile metabolites and/or other antimicrobials (Kinderlerer, 1995; Leong *et al*., 2011). Strain FRR 0530 exhibits keratinolytic activity and is considered potentially pathogenic towards humans and animals (Kushwaha, 1980; Marchisio *et al*., 1986).

***Strains within the Aspergillus* : *Penicillium* : *Eurotium* clade**

*Aspergillus penicillioides* Antique wooden Hallsworth laborat- Studies of xerophilicity have demonstrated mycelial extension down to 0.647 water

JH06GBM chopping-block, ory (Queen’s activity on media supplemented with glycerol+NaCl+KCl (Williams and Hallsworth,

made from syca- University Belfast) 2009). Water : temperature relations have been characterized on glycerol-

more (UK, 2006) supplemented media (Williams and Hallsworth, 2009). *A.* *penicillioides* JH06GBM,

JH06THH, and FRR 2179 were used in studies which provided evidence that hydrophobic substances induce cellular water stress via a chaotropicity-mediated mode-of-action: compatible solute synthesis/accumulation occurred in mycelium on media supplemented with sucrose+octanol (Bhaganna et al., 2010). Conidia tenacity of this strain was studied to determine interactions between the chao- or kosmotropic solutes and exposure to extreme temperatures and pressure (Chin *et al*., 2010).

*Aspergillus penicillioides* Antique wooden Hallsworth laborat- Studies of xerophilicity have demonstrated mycelial extension down to 0.647 (Will-

JH06THJ artifact (Thailand, ory (Queen’s iams and Hallsworth, 2009) and 0.640 water activity on media supplemented with

2006) University Belfast) glycerol+NaCl+KCl and glycerol+glucose+fructose, respectively (Stevenson *et al*.,

2015). Water : temperature relations have been characterized on glycerol-supplemented media (Williams and Hallsworth, 2009)

*Aspergillus penicillioides* Antique wooden Hallsworth laborat- Studies of xerophilicity have demonstrated mycelial extension down to 0.647 water

JH06THH artifact (Thailand, ory (Queen’s activity on media supplemented with glycerol+NaCl+KCl (Williams and Hallsworth,

2006) University Belfast) 2009). Water : temperature relations have been characterized on glycerol-

supplemented media (Williams and Hallsworth, 2009). *A.* *penicillioides* JH06GBM, JH06THH, and FRR 2179 were used in studies which provided evidence that hydrophobic substances induce cellular water stress via a chaotropicity-mediated mode-of-action: compatible solute synthesis/accumulation occurred in mycelium on media supplemented with sucrose+octanol (Bhaganna et al., 2010).

*Eurotium amstelodami* Dates; *Phoenix* ATCC 16464 Studies of xerophilicity have demonstrated mycelial extension down to 0.656 water

FRR 2792 *dactylifera* (country CBS 518.65 activity on media supplemented with glycerol+sucrose+NaCl+KCl (Williams and

not recorded, 1910) IAM 13827 Hallsworth, 2009). Water : temperature relations have been characterized on

IMI 229971 glycerol-supplemented media (Williams and Hallsworth, 2009). Strain FRR 2792

NBRC 33018 *was* used in studies which provided evidence that hydrophobic substances induce

cellular water stress via a chaotropicity-mediated mode-of-action: compatible solute synthesis/accumulation occurred in mycelium on media supplemented with sucrose+octanol (Bhaganna et al., 2010). FRR 2792 is a well-characterized strain that is frequently employed in phylogenetic studies (Tamura *et al*., 1999; Peterson, 2008; Houbraken and Samson, 2011; Pettersson *et al*. 2011)

*Eurotium echinulatum* Sultanas (Australia, None There is a paucity of studies on strain FRR 5040, however studies of xerophilicity

FRR 5040 1997) have demonstrated germination of conidia of *E. echinulatum* Delacroix. (FRR 1661)

at 0.620 water activity after two years (Snow, 1949), the lowest water activity hitherto reported for biotic activity of any *Eurotium*. Delacroix (1893a, 1893b) described the anamorph and teleomorph of this species as *Aspergillus brunneus* and *E. echinulatum*; *A. brunneus* is synonymous with *Aspergillus echinulatus* (Hubka *et al*., 2013)

*Eurotium halophilicum* Cardamom seeds ATCC 62923 Studies of xerophilicity have demonstrated germination and hyphal growth down to

FRR 2471 (Australia, 1982) CBS 398.87 0.675 water activity on media supplemented with glucose+fructose (Andrews and

IMI 313775 Pitt, 1987; Hocking and Pitt, 1988)

*Eurotium repens*  Antique wooden Hallsworth laborat- Studies of xerophilicity have demonstrated mycelial extension down to 0.667 water

JH06JPD rice-scoop (Japan, ory (Queen’s activity on media supplemented with glycerol+sucrose+NaCl+KCl (Williams and

2006) University Belfast) Hallsworth, 2009). Water : temperature relations have been characterized on

glycerol-supplemented media (Williams and Hallsworth, 2009). Conidia tenacity of this strain was studied to determine interactions between the chao- or kosmotropic solutes and exposure to extreme temperatures and pressure (Chin *et al*., 2010)

___________________________________________________________________________________________________________

a. The culture collections in which strains are deposited included are: the Division of Food Research, Food Research Laboratory, CSIRO, Australia (FRR), the American Type Culture Collection, Virginia, USA (ATCC), Centraalbureau voor Schimmelcultures, Utrecht, The Netherlands (CBS), CABI Bioscience, Eggham, UK (IMI), NITE Biological Resource Center, Department of Biotechnology, National Institute of Technology and Evaluation, Kisarazu, Japan (NBRC), and University of Alberta Mold Herbarium and Culture Collection, Edmonton, Canada (UAMH). *Aspergillus penicillioides* strains JH06GBM JH06THH, and JH06THJ and *Eurotium repens* strain JH06JPD are currently held at the Hallsworth laboratory (Queen’s University Belfast, UK) and are available from the corresponding author upon request.

**Table S2**. Media used for germination assays^a^

**Water activity**^b^  **pH Chao-/kosmo- Stressor type and concn. (M)**

**tropicity (kJ g^-1^)**

0.765 6.7 1.41 glycerol (5.50) + NaCl (0.50)

0.741 6.8 0.66 glycerol (5.50) + NaCl (1.00)

0.734 6.6 12.45 glycerol (5.50) + sucrose (0.25)

0.709 6.7 -4.15 glycerol (5.50) + NaCl (1.50)

0.707 6.3 18.43 glycerol (7.00)

0.701 6.7 1.11 glycerol (5.50) + NaCl (0.50) + sucrose (0.30)

0.699 6.5 10.95 glycerol (5.50) + sucrose (0.50)

0.694 5.6 18.92 glycerol (5.50) + glucose (0.80) + fructose (0.80)

0.692 6.8 -5.15 glycerol (5.50) + NaCl (1.60)

0.685 6.7 -0.62 glycerol (5.50) + NaCl (0.50) + sucrose (0.50)

0.674 6.5 9.13 glycerol (5.50) + sucrose (0.65)

0.668 6.8 -6.49 glycerol (5.50) + NaCl (1.70)

0.664 6.3 19.59 glycerol (7.10)

0.654 6.2 21.58 glycerol (7.20)

0.651 6.6 -1.12 glycerol (5.50) + NaCl (0.80) + sucrose (0.50)

0.649 5.4 20.75 glycerol (5.50) + glucose (1.00) + fructose (1.00)

0.647 6.2 22.36 glycerol (7.30)

0.640 6.7 -11.04 glycerol (5.50) + NaCl (1.80)

0.639 7.0 -2.14 glycerol (5.50) + NaCl (0.80) + sucrose (0.50) + KCl (0.20)

0.637 6.3 7.30 glycerol (5.50) + sucrose (0.80)

0.635 6.2 22.83 glycerol (7.40)

0.628 6.7 -12.45 glycerol (5.50) + NaCl (1.90)

0.623 6.5 -4.31 glycerol (5.50) + NaCl (1.00) + sucrose (0.70)

0.621 6.2 26.56 glycerol (7.50)

0.619 6.3 5.39 glycerol (5.50) + sucrose (0.95)

0.611 5.4 22.74 glycerol (5.50) + glucose (1.25) + fructose (1.25)

0.608 6.1 27.64 glycerol (7.60)

0.605 6.5 -14.94 glycerol (5.50) + NaCl (2.00)

0.602 6.8 -5.38 glycerol (5.50) + NaCl (0.80) + sucrose (0.80) + KCl (0.20)

0.601 6.3 2.69 glycerol (5.50) + sucrose (1.10)

0.598 6.3 -6.52 glycerol (5.50) + NaCl (1.20) + sucrose (0.80)

0.592 5.3 25.49 glycerol (5.50) + glucose (1.50) + fructose (1.50)

0.591 6.2 0.65 glycerol (5.50) + sucrose (1.30)

0.585 6.1 29.05 glycerol (7.70)

0.579 7.0 -6.68 glycerol (5.50) + NaCl (1.00) + sucrose (0.80) + KCl (0.20)

0.575 6.6 -24.90 glycerol (5.50) + NaCl (2.30)

_________________________________________________________________________________________

^a^All media were based on MYPiA (Williams and Hallsworth, 2009), and contained: 1% malt extract, 1% yeast

extract, 0.1% KH_2_PO_4_, and 1.5% (w/v) agar.

^b^The water activity of each medium was measured at 30ºC, and replicate values were within ±0.002 water

activity (see *Experimental procedures*).

**Fig. S1:**

**Supporting references**

Delacroix G. (1893a). Champignons parasites nouveaux. *Bull Soc Mycol Fr* **9:** 264–268.

Delacroix G. (1893b). Espèces nouvelle observées au Laboratoire de Pathologie vegetale. *Bull Soc Mycol Fr* **9:** 184–188.

Hocking AD, Pitt JI. (1988). Two new species of xerophilic fungi and a further record of *Eurotium halophilicum*. *Mycologia* 82–88.

Kinderlerer, J.L. (1987) Ethanol production in table jelly by two species of *Chrysosporium*. *J Appl Bacteriol* **63**: 395–399.

Kinderlerer, J.L. (1995) Czapek Casein 50% Glucose (CZC50G): a new medium for the identification of foodborne *Chrysosporium spp*. *Lett Appl Microbio* **21**: 131–136.

Kinderlerer, J.L. (1997) *Chrysosporium* species, potential spoilage organisms of chocolate. *J Appl Microbiol* **83**: 771–778.

Kushwaha, R.K.S. (1980) The genus *Chrysosporium*, its physiology and biotechnological potential. *Stud Mycol* **20**: 66–76.

Leong, S.L., Pettersson, O.V., Rice, T., Hocking, A.D., Schnürer, J. (2011). The extreme xerophilic mould *Xeromyces bisporus* – growth and competition at various water activities. *Int J Food Microbiol* **145**: 57–63.

Marchisio, V.F. (1986) Keratinolytic and keratinophilic fungi of children's sandpits in the city of Turin. *Mycopathologia* **94**: 163–172.

Park, H.G., Jong, S.C. (2003) Molecular characterization of *Monascus* strains based on the D1/D2 regions of LSU rRNA genes. *Mycoscience* **44**: 25 –32.

Peterson, S.W. (2008). Phylogenetic analysis of *Aspergillus* species using DNA sequences from four loci. *Mycologia* **100**: 205–226.

Tamura, M., Kawasaki, H., Sugiyama, J. (1999) Identity of the xerophilic species *Aspergillus penicillioides*: Integrated analysis of the genotypic and phenotypic characters. *J Gen Appl Microbiol* **45**: 29–37.
